# Abdominal Abscesses and Destruction of Inguinal Canal with Mesh Dislocation caused by a Perforated Diverticulitis

**DOI:** 10.1155/2019/8049393

**Published:** 2019-10-29

**Authors:** Christoph Paasch, Franziska Renger, Sergej Baschinskij, Martin W. Strik

**Affiliations:** ^1^Department of General, Visceral and Cancer Surgery, Helios Klinikum Berlin-Buch, Schwanebecker Chaussee 50, 13125 Berlin, Germany; ^2^Institute of Pathology, Helios Klinikum Emil von Behring, Walterhöferstraße 11, 14165 Berlin, Germany

## Abstract

The diverticulitis is a frequent disease of the gastrointestinal tract. It may lead to a variety of severe complications. In some cases, it has to be surgically treated. Herein, we present a rare case of a 66-year-old man, who suffered from a painful, visible “fist sized” mass of the left lower abdomen. A perforated diverticulitis with abdominal, cutaneous abscesses and destruction of the inguinal canal with mesh dislocation was diagnosed and successfully surgically treated.

## 1. Introduction

The diverticulitis is a frequent disease of the gastrointestinal tract with a prevalence of 28-45% in the population. Mostly, the colon sigmoideum (CS) is affected. Patients suffering from a diverticulitis should receive a “stage-related” therapy according to the national and international guidelines [1]. In some cases, the diverticulitis leads to rare and severe complications like in the case report at hand.

## 2. Case Presentation

A male patient, 66 years of age, was referred to our hospital in December 2018. He suffered from pain of the left lower abdomen, night sweat, and intestinal spasms since three weeks. These symptoms mainly appeared at night. In these time period, our patient lost 5 kilogram of weight. Six years ago, the patient underwent a coloscopy. Diverticular disease was diagnosed. The family anamnesis was negative in terms of cancer and hereditary diseases. His medical history included an arterial hypertonus, auricular fibrillation, coronary heart disease, diabetes II, and hypothyreosis. The patient underwent radical prostatectomy due to a prostate carcinoma (2013). Moreover, he received an inguinal hernia repair in Shouldice technique in 2000. A hernia relapse was treated with hernia repair with a transabdominal preperitoneal approach (TAPP) with placement of a polypropylene mesh (10 × 15 cm) one year later at our hospital. The mesh was not fixated.

**Figure 1:**
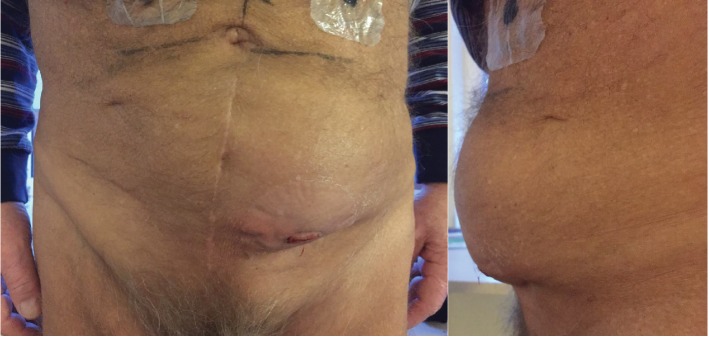
The picture shows a “fist sized” mass of the left lower abdomen.

The clinical examination revealed a palpable, painful, and visible mass of the left lower abdomen with inflammation of the skin (Figure 1).

**Figure 2:**
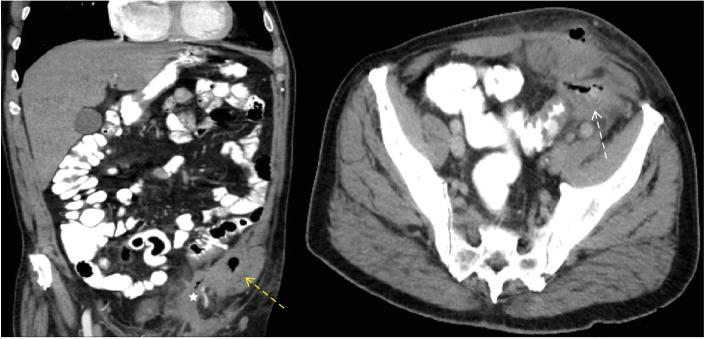
Computed tomography of the abdomen; the picture highlights an inflamed 4.6 × 9.5 cm sized conglomerate adjacent to the abdominal wall and the left inguinal canal (yellow and white dashed arrow). The prior implanted mesh seemed to be partially dislocated to the abdominal cavity (white star).

The laboratory test yielded slightly elevated blood levels of C-reactive protein and leucocytes. The patient received an ultrasound imaging of the abdomen. A 5 × 8 cm sized abscess of the left lower abdomen was diagnosed. We therefore conducted a computed tomography of the abdomen. The examination detected an inflamed 4.6 × 9.5 cm sized conglomerate mass adjacent to the abdominal wall and the left inguinal canal (Figure 2). The prior implanted mesh seemed to be partially dislocated to the abdominal cavity.

An inflamed stenosis of CS was diagnosed, when we conducted a coloscopy.

**Figure 3:**
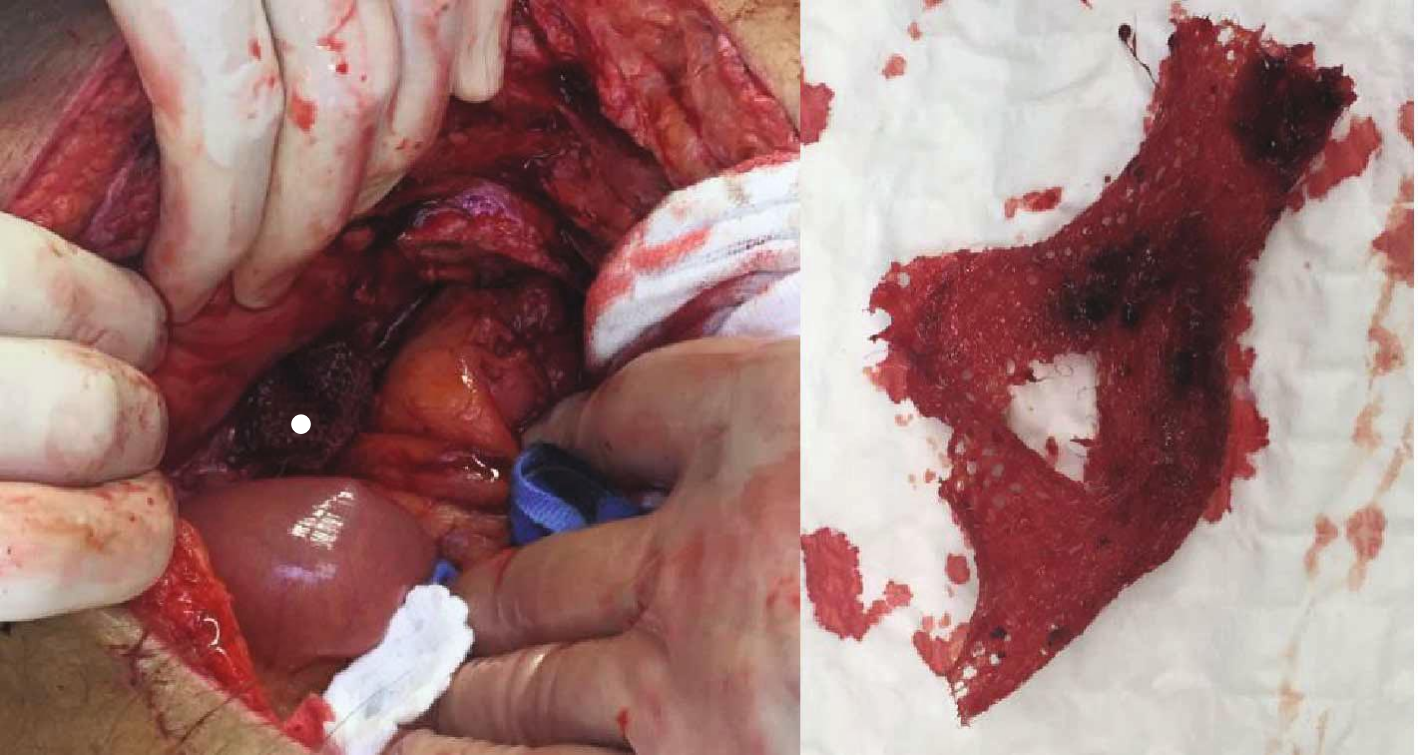
The picture shows the opened left inguinal canal with the mesh (white point). On the right side of the image, the mesh has been removed.

**Figure 4:**
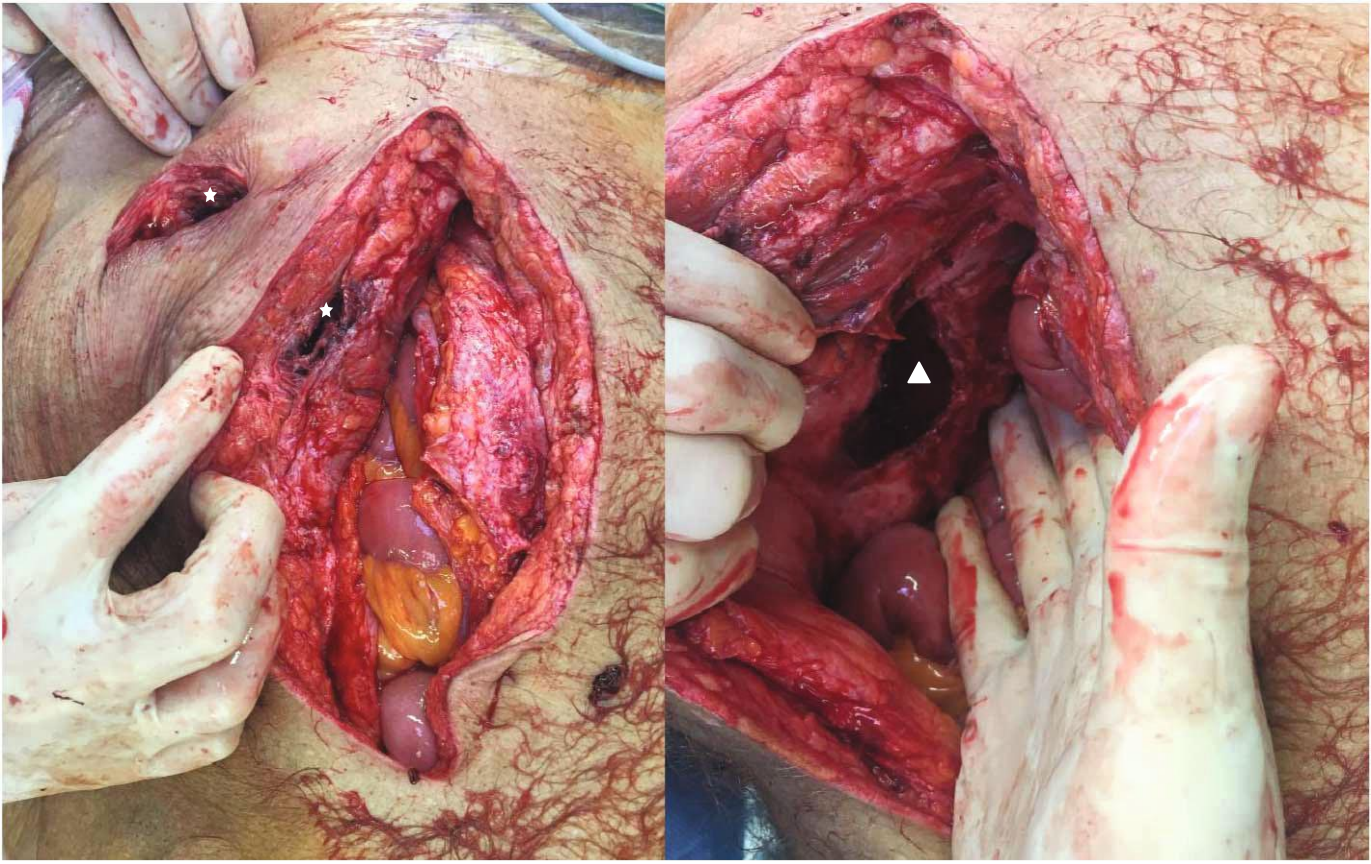
The subcutaneous and cutaneous opened abscess cavity is indicated by the white star. This cavity was connected with the left inguinal canal (white triangle).

Under suspicion of a colonic diverticular abscess, the patient underwent surgery. Explorative laparotomy exposed a perforated diverticulitis of CS adjacent to the abdominal wall and with inflammatory destruction of the inguinal canal and mesh dislocation (Figures 3 and 4). After abscess and mesh removal with consecutive abdominal lavage, we resected CS with primary stapled anastomosis as descendorectostomy. The inguinal defect was sutured. Due to the peritonitis, a mesh was not placed.

As expected, the histological examination of the removed CS revealed a perforated diverticulitis without any sign of malignancy.

The postoperative course was uneventful, and the patient left our hospital 11 days after surgery.

## 3. Discussion

The case report describes a very rare course of an acute complicated diverticulitis and mesh migration. The diverticulitis is a frequent disease of the gastrointestinal tract. The most common complications include perforation with or without fecal peritonitis, stenosis, abscesses, fistulas, and endoluminal bleeding [1]. Nevertheless, in literature, untypical clinical courses and manifestations have been described. Simillis et al. published a case of small bowel obstruction secondary to mesh erosion [2]. Also, composite mesh migration into the colon has been previously described [3, 4]. Moreover, a diverticulitis can cause a pylephlebitis, which may lead to ubiquitous abscesses [5]. Kaiser et al. [6] treated a patient who suffered from a solitary liver abscess caused by a severe diverticulitis [6]. In addition, Valero et al. [7] published a case report of a patient, who suffered from cerebral abscesses [7]. Also, a pyogenic ventriculitis as clinical presentation of a diverticulitis has been described in literature [8]. Moreover, a diverticulitis may lead to colourethral as well as colouterine fistulas [9]. In some cases, foreign material has to be explanted. To that, Varmeulen et al. (2012) treated a 59-year-old man, who suffered from dorsal pain. A computed tomography of the abdomen revealed a diverticulitis with fistulas to the bladder and to a prior implanted neurostimulator. The authors performed a two-stage procedure with drainage of the abscess, removal of the *corpus alienum* followed by a sigmoid resection one week later [10].

With appropriate intraoperative images, the case report at hand describes a rare clinical course of a complicated diverticulitis, especially in terms of the inguinal canal destruction with mesh dislocation and the “fist sized” palpable abdominal conglomerate. Most likely, the immunodeficient condition of our patient caused by his multimorbidity with arteriosclerosis and diabetes II leads to these severe clinical courses [11]. To summarize, it is possible that a contained perforation of diverticulitis led to tissue disruption near the inguinal canal and cause mesh migration. On the other hand, mesh migration could have led to erosion, and contained perforation of sigmoid diverticulitis, which manifested as a pericolonic abscess. We assume that the contained perforation of diverticulitis led to the tissue disruption. On the one hand, the TAPP procedure was performed already 14 years ago with standardized closure of the peritoneum by suture. This prevented bowl contact. One could argue that the nonfixated mesh led to migration. The question of the need for fixation in laparoscopic inguinal hernia repair with mesh has been investigated previously. In laparoscopic groin hernia repairs, nonfixation of mesh is recommended by the HerniaSurge Group with the exception of large medial defects [12, 13]. It has to be mentioned that due to lack of documentation, we are not able to reveal whether our patient had a large medial hernia or not.

In terms of the management of an infected or contaminated mesh after inguinal hernia repair, there are no evidence-based guidelines. The optimal duration for the treatment of persistent mesh infection as conservative management (donation of antibiotic and drainage placement) remains unclear. The complete mesh excision is described as the final solution for cases with extensive mesh infection like in the case report at hand [14]. Further clinical trials are mandatory to reveal more evidence on that topic.

## 4. Conclusion

Diverticulitis leads to severe complications. It may cause an inflamed abdominal conglomerate mimicking a malignant tumour.

When diagnosing a palpable and painful abdominal mass, a complicated diverticulitis should be taken under consideration as differential diagnosis.
